# Supplementary material for: Psychomotor and visual skills underlying position specialization in 1352 elite youth baseball players
Source: PLoS One. 2023 Jan 19;18(1):e0278689. doi: 10.1371/journal.pone.0278689 (PMC9851526; doi:10.1371/journal.pone.0278689)
Supplement: Attachment — Submitted filename: Review comment.docx [file pone.0278689.s001.docx]

**Review comment**

This manuscript entitled “Psychomotor and Visual Skills Underlying Position Specialization in 1352 Elite Youth Baseball Players” primarily evaluated data from 1352 elite junior male PDP participants (aged 14 to 21), to identify latent abilities and their association with player specialization. This research These findings indicate skills that contribute to player specialization, providing new information about the developmental trajectory of junior elite baseball athletes. But there are several questions that should be addressed before this manuscript can be accepted for publication, which lists below. You can revise this paper more properly. I suggest that you improve the description below.

**Specific comments**

1. The Introduction section introduces the purpose and significance of this research in more detail, but lacks definitions of related concepts, such as Psychomotor and Visual Skills. In addition, the Introduction section has to explain why Psychomotor and Visual Skills in baseball are important research questions.
2. In the introduction section, there is some sentence that has to add the reference. Such as “The physical and mental demands of the sport are so great that a player who can get on base one out of every three plate appearances is considered among the best-of-the-best on offense” and “On defense players are separated into nine positions that can be grouped into four broader categories: pitchers, catchers, infielders, and outfielders.”
3. In the sample selection section, please describe more details about the excluded condition of the sample.
4. Do subjects need to do preparatory activities before being formally tested? Experiment-based reproducibility principle, please show more details.
5. In the statistical analysis section, please described the details of measures for possible interactions in repeated measures analysis.
6. This study divided the subjects into four groups (catchers, infielders, outfielders, and pitchers), please provide more details to explain the basis for the grouping.
