# Supplementary material for: Psychomotor and visual skills underlying position specialization in 1352 elite youth baseball players
Source: PLoS One. 2023 Jan 19;18(1):e0278689. doi: 10.1371/journal.pone.0278689 (PMC9851526; doi:10.1371/journal.pone.0278689)
Supplement: Attachment — Submitted filename: Review comment.docx [file pone.0278689.s002.docx]

**Review comment**

Congratulations to the authors for the interesting work. The research is original and I enjoyed reading it. I suggest minor comments in order to improve the quality of the manuscript.

**Specific comments**

1. The structure of this manuscript is somewhat complex, and to help the reader feel more comfortable reading this study, I suggest adding a new paragraph at the end of the introduction section with a brief description of the structure of this manuscript.
2. L125-142. The description of the test process section needs to add further details, such as the requirements for athletes during the test, whether there is an interval between each test, etc. A detailed description of the test procedure is necessary for the principle of reproducibility in clinical trials.
3. L201-205. In a formal experiment, do subjects get only one chance to be tested? Will the test be allowed to be repeated if the subject makes a mistake? Please add specific details.
4. Were possible interaction issues during testing reported in the results section of this study?
5. L316-320. Please check this section for formatting issues and the header integrity of Table 1.
